# Low-temperature synthesis of carbon nanotubes on indium tin oxide electrodes for organic solar cells

**DOI:** 10.3762/bjnano.3.60

**Published:** 2012-07-19

**Authors:** Andrea Capasso, Luigi Salamandra, Aldo Di Carlo, John Marcus Bell, Nunzio Motta

**Affiliations:** 1 School of Chemistry Physics and Mechanical Engineering, Queensland University of Technology, George St, 4000 Brisbane, Australia; 2 CHOSE (Centre for Hybrid and Organic Solar Energy), Department of Electronic Engineering, University of Rome Tor Vergata, Via del Politecnico 1, 00133 Rome, Italy

**Keywords:** carbon nanotubes, electrode, indium tin oxide, Kelvin probe, organic photovoltaics

## Abstract

The electrical performance of indium tin oxide (ITO) coated glass was improved by including a controlled layer of carbon nanotubes directly on top of the ITO film. Multiwall carbon nanotubes (MWCNTs) were synthesized by chemical vapor deposition, using ultrathin Fe layers as catalyst. The process parameters (temperature, gas flow and duration) were carefully refined to obtain the appropriate size and density of MWCNTs with a minimum decrease of the light harvesting in the cell. When used as anodes for organic solar cells based on poly(3-hexylthiophene) (P3HT) and phenyl-C61-butyric acid methyl ester (PCBM), the MWCNT-enhanced electrodes are found to improve the charge-carrier extraction from the photoactive blend, thanks to the additional percolation paths provided by the CNTs. The work function of as-modified ITO surfaces was measured by the Kelvin probe method to be 4.95 eV, resulting in an improved matching to the highest occupied molecular orbital level of the P3HT. This is in turn expected to increase the hole transport and collection at the anode, contributing to the significant increase of current density and open-circuit voltage observed in test cells created with such MWCNT-enhanced electrodes.

## Introduction

Following the original proposal for the creation of plastic solar cells [1], many research efforts have been recently directed to improve their power-conversion efficiency (PCE), in order to make these cells commercially viable [2]. The most promising active materials for organic cells are semiconducting polymers and fullerene derivatives, whose mixtures result in the formation of an interpenetrated phase consisting of nanoscaled bulk heterojunctions [1]. High performance has been predicted theoretically for these devices, which are characterized by low processing costs and mechanical flexibility [3], making them particularly attractive in comparison to those based on crystalline silicon and on other expensive inorganic semiconductors.

At present, the most successful and widespread blend for organic photovoltaics is based on a composite of poly(3-hexylthiophene) (P3HT) and phenyl-C_61_-butyric acid methyl (PCBM) [4–5]. In this cell architecture the polymer acts as an electron donor and the fullerene derivative acts as an electron acceptor [6]. The holes move in the polymeric phase towards the anode, while the electrons hop along the fullerenes and eventually reach the cathode. Since the diffusion length of the exciton in the polymers is very low, recombination is highly probable, unless the electron is quickly injected into the carbon (acceptor) phase. Unfortunately, the concurrence of a low electrical mobility (due to the hopping mechanism) with a small exciton diffusion length increases the likelihood of charge recombination, ultimately affecting the overall PCE of the cells [7]. Many approaches have been proposed in order to overcome such fundamental issues and to improve the performances of P3HT:PCBM solar cells. In particular, very promising advances can be gained by increasing the nanoscale ordering of the polymer/fullerene composite. Different means have been proposed, such as thermal [8] and solvent annealing [9], or the use of additives in the blend preparation [10].

Along with fullerenes, carbon nanotubes (CNTs) have also been suggested as promising materials to boost solar cell PCE, thanks to their excellent electrical properties and to a favorable aspect ratio [11]. In fact, CNTs were initially suggested as a replacement for fullerene [12], because of their ability to create percolation paths through the heterostructure, while providing electron–hole dissociation sites. Being that the electron mobility in fullerenes is rather low [13–15], the initial motivation for the replacement of PCBM with CNTs was an expected increase in electron mobility due to ballistic transport in the CNT phase. Besides, microscopic studies proved that in a mixture of P3HT and CNTs, the polymer self-assembles and wraps the carbon nanostructure, generating a bulk heterojunction with a large interface area over which a strong electric field would lead to a high probability of exciton dissociation [16]. However, the lack of control over the selection of the CNTs has made their integration with polymers quite unsuccessful so far [17], as recently suggested by photoluminescence studies [18]. In fact, when P3HT is mixed with both semiconducting and metallic CNTs, the latter tend to create Schottky barriers [16,19] at the polymer–CNT interface, which can favor the electron–hole recombination and thus decrease the short-circuit current. This has been also confirmed by Valentini et al. [20], who were able to attain a marked increase in the short-circuit current of the cell by depositing only semiconducting CNTs on the ITO surface. Nevertheless, this situation is still under debate, since efficient electron–hole separation has been recently observed in P3HT mixed with metallic and semiconducting CNTs [21], suggesting that both kinds of nanotubes could ultimately act as hole acceptors.

Whatever the solution to this puzzle is, including MWCNTs in a blend of P3HT and PCBM matches the key objective of achieving large interfacial areas within a bulk donor–acceptor heterojunction mixture, as proposed by Berson et al. [22]. As metallic conductors, MWCNTs are expected to lower the electrical percolation threshold even at minimal concentrations, due to their high electron conductivity and their shape. Following this line, Sun [2] proposed the assembly of a cell containing a network of vertically aligned CNTs separated by vertical polymer layers. The realization of this idea would grant a major increase in conductivity at the electrode (thanks to the interpenetrating structure of vertically aligned nanotubes), but it has not been completely exploited so far, because of its intrinsic complexity. Nonetheless, as a first attempt in this direction, Miller et al. [23] reported the synthesis of CNTs directly on ITO–glass by chemical vapor deposition (CVD). Although successful in terms of CNT yield, their method did not provide a specific control over the assembly of the CNTs, whose high density rendered the ITO electrode almost opaque. Conversely, in this paper we present the first evidence of the controlled growth of MWCNTs on ITO electrodes, obtained by a fine tuning of the CVD parameters, such as temperature, gas flow, and duration. By selecting the optimal combination of these parameters it is possible to create MWCNT mats with the required size and density on the ITO-coated glass surface. Such CNT-enhanced electrodes are found to show advantages in terms of work-function (WF) matching and electrical properties in comparison with pristine ITO electrodes, contributing to the significant advancement of the overall PCE of the solar cell.

## Results and Discussion

**Figure 1:**
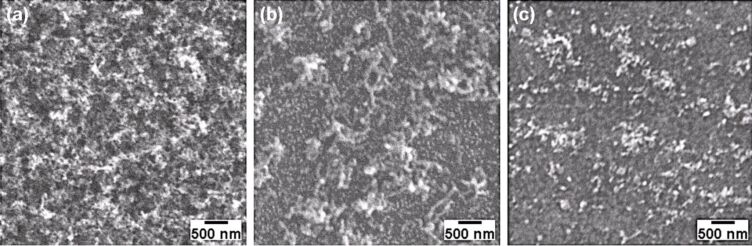
SEM images of MWCNTs grown on ITO-coated glass by CVD at: (a) 550 °C, (b) 525 °C, (c) 500 °C.

After preliminary tests in CVD, SEM and EDX analysis indicated that the range of temperature of 550–600 °C has to be avoided for the application of the ITO-substrates as electrodes, since the ITO layer undergoes severe disruption at such high temperatures, becoming no longer conductive. The growth time was also inspected, determining an optimal CNT synthesis time of 30 min. Successful growth of MWCNTs was obtained on Samples A, B and C, treated in CVD for 30 min at 550, 525 and 500 °C respectively (Figure 1).

The transmittance and the resistivity of each electrode were measured and are reported in Table 1. The density distribution of the tubes is found to scale considerably with the deposition temperature. On Sample A (550 °C), a dense and thick layer of MWCNTs covers the entire ITO area. Due to the CNT density, the substrate looks almost black and is therefore no longer transparent. In addition, the sheet resistance of the ITO layer increased to 40 Ω/sq (almost three times higher than that for pristine ITO). When heated in air at 550 °C, the polycrystalline ITO layer is known to degrade, leading to the segregation of Sn into clusters hundreds of nanometers in diameter [24], and also to increased inter-diffusion between the substrate and film [25]. Both effects can reduce the film conductivity by up to 50%, as reported elsewhere [26].

**Table 1:** Growth parameters and properties for the three CNT-enhanced electrodes compared to pure glass and to ITO/glass sample.

sample	growth *T* [°C]	R [Ω/sq] ITO film	transmittance at 510 nm [%]

glass	—	—	89
glass/ITO	—	15	81
Sample C (glass/ITO+CNT)	500	25	75
Sample B (glass/ITO+CNT)	525	33	45
Sample A (glass/ITO+CNT)	550	40	0

In our case, the interaction of the ITO film with the CVD process gases at 550 °C is expected to deteriorate the conductivity of the electrode even more strongly. This is partly supported by the formation of microspheres of indium on the ITO film, as observed by SEM and EDX (not shown). Similarly to what was reported by Lan et al. [27], we suggest that the exposure of the ITO film to a hydrogen atmosphere at 550 °C (and the probable creation of atomic hydrogen coming from the dissociation of either H_2_ or C_2_H_2_, perhaps enabled by the metal catalyst layer) enables the formation of small clusters of metallic indium, which coalesce during the CVD to form spherical particles with a typical size of >2 µm. As a consequence, the film surface would segregate and change its chemical ratio. The film conductivity will in turn significantly decrease, as will the optical transmittance, on account of a stronger light absorption and scattering caused by those metallic microspheres.

**Figure 2:**
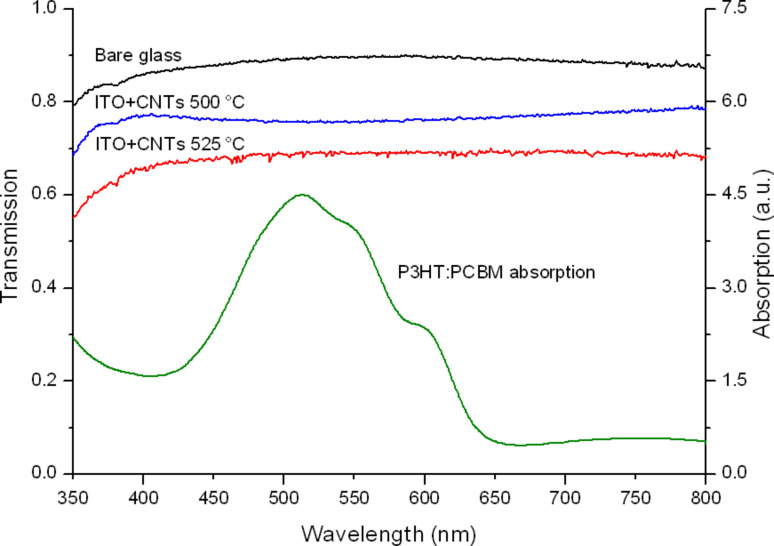
Transmittance spectra of the electrodes (left Y axis), compared to the absorption spectrum of the P3HT:PCBM blend (right Y axis).

In contrast, at 525 °C (Sample B) and 500 °C (Sample C), the degradation is not as severe and the conductivity of the film is still acceptable (25–30 Ω/sq). In these two cases the nanotubes nucleate with a lower density, and the substrates show a transmittance at 515 nm of 45 and 75%, respectively. Figure 2 illustrates the optical transmittance of these two samples in the wavelength range of 350–750 nm, taking also into consideration the absorption spectrum of the P3HT:PCBM blend.

**Figure 3:**
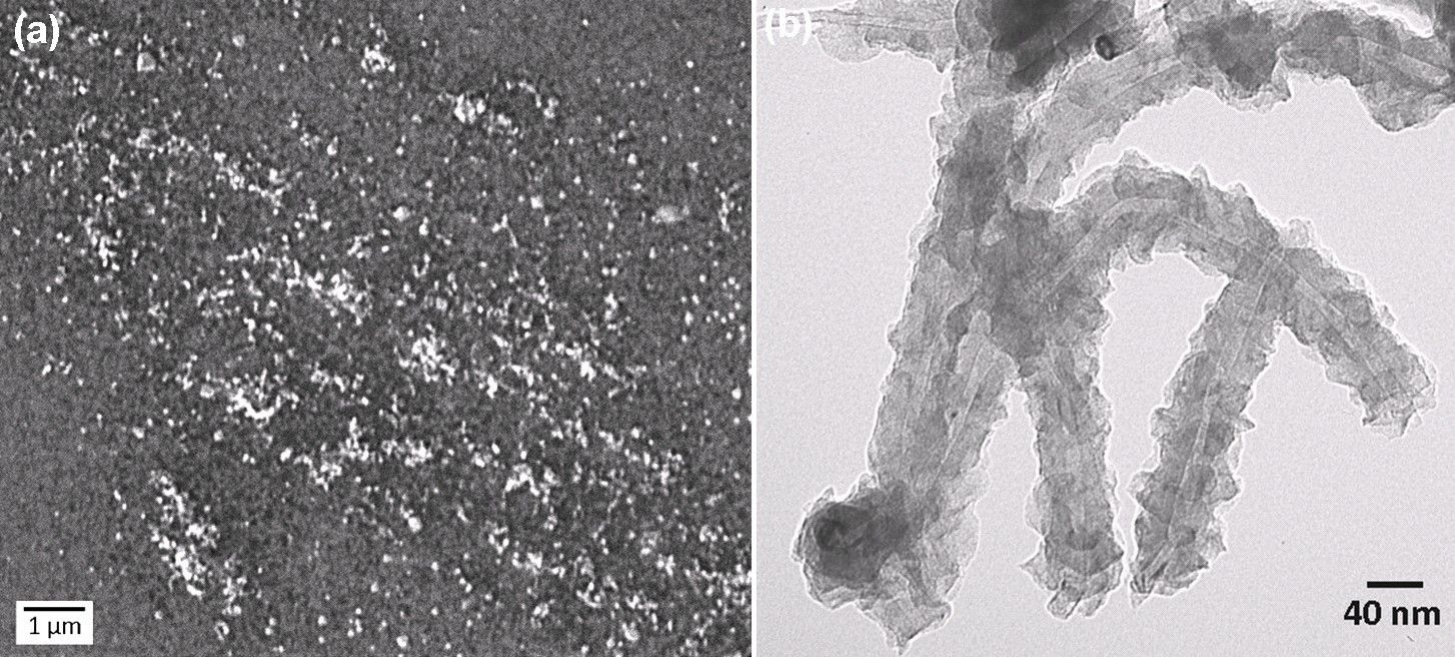
(a) SEM image showing the surface of Sample C, on which a low density mat of MWCNTs is grown after CVD at 500 °C for 30 min. (b) TEM image of CNTs from Sample B (grown in CVD for 30 min at 525 °C).

Although on Sample B the density of the CNT carpet is much higher than on Sample C, for the present purpose, an optimal condition is reached with the latter sample. When a temperature of 500 °C is used, the short sparse tubes (average density of 10 tubes/µm^2^) that grow do not form bundles or thick aggregates, allowing more light to pass through the electrode and to reach the active layer of the cell. SEM images taken in various sites of Sample C (as the one in Figure 3a) were analyzed to calculate the average dimensions of the grown MWCNTs. The average length of the tubes is 100 nm and the diameter 40 nm, as confirmed by TEM analysis (Figure 3b). Due to the low synthesis temperature the tube structure is very defective and residual allotropes of carbon, such as diamond-like and amorphous carbon, are found around the nanotube walls (confirmed also by Raman spectroscopy, not shown).

In our context, the presence of defects in the tubular structure could be an advantage in terms of conductivity, because it can induce cross linking between the inner shells (walls) of the tubes through sp^3^ bond formation, facilitating charge-carrier hopping to inner shells [28]. Such intershell bridging provides additional charge-carrier transport pathways, offsetting the effect of a conductivity decrease induced by defect scattering.

By measuring various areas of the sample, a mean distribution of 10 MWCNT/µm^2^ has been estimated, being that the average dimensions of nanotubes are 100 nm in length, 30 nm in diameter, and 58 m^2^/g for the specific surface area [29]. Such values would entail an increase of 10% in the overall surface area of the CNT/ITO electrode in comparison with the planar ITO film. We believe that such three-dimensional and nanostructured electrodes, made of metallic nanotubes [30], will be able to penetrate the P3HT:PCBM blend and ease the extraction of holes to the external circuit.

Using Sample C, we measured the WF of the as-created electrode. Kelvin probe and ultraviolet photoelectron spectroscopy (UPS) are the techniques usually employed for this purpose; however, there are substantial differences in how the WF is measured. The Kelvin probe method measures, in air, the difference in WF between a millimetric probe and the sample, which can undergo surface reactions with species adsorbed from the environment. Conversely, UPS measures, in ultrahigh vacuum, the lowest WF of a small portion of the surface, usually a few microns in diameter. WF values measured by the Kelvin probe method are often higher than those measured by UPS [31], due to the influence of the ambient gases and to the fact that the probe size typically covers an area of few millimeters squared. Therefore, we chose to use the Kelvin probe method as it is able to measure the electrode WF in its working environment, just before the cell is built.

After a fine calibration with a reference tantalum foil, the WF of an untreated and clean ITO substrate was found to be 4.80 eV. We then measured a value of 4.95 eV in the case of our CNT-enhanced electrode, that is, an increase of 0.15 eV. Although this value is in good agreement with the WF of MWCNTs reported by Shiraishi et al. [32], we have to make two considerations: (i) our substrate is not fully covered by a continuous, dense mat of nanotubes; (ii) when measuring by Kelvin probe method, the electrode under test is the whole structure CNT/ITO, not only the CNT overlayer; (iii) a thin layer of Fe is also present between the ITO and the CNT layer, even if during the CVD it should become segregated into small particles, giving rise to the nucleation of the tubes.

**Figure 4:**
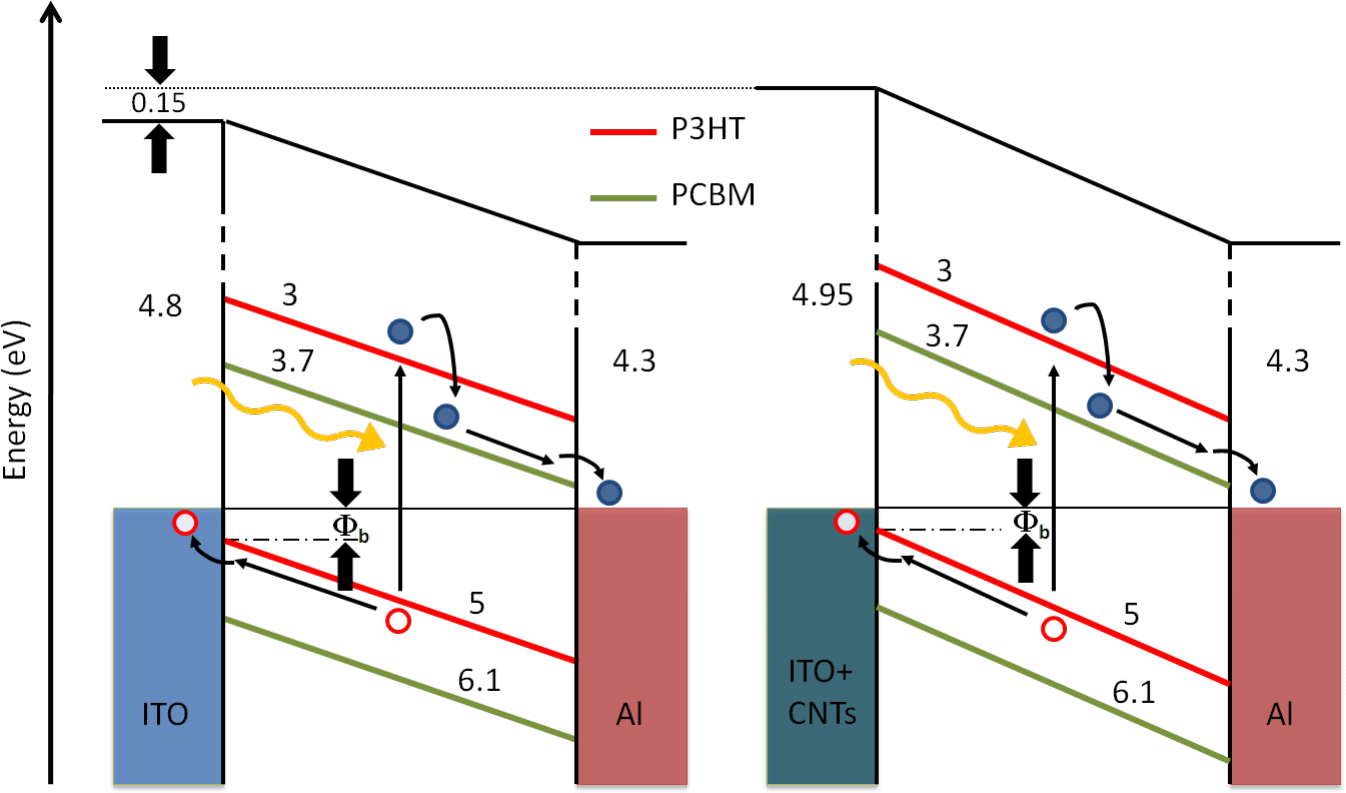
WF levels for cells with ITO (left) and ITO–CNT (right) electrode. (All reported values are in eV and negative).

All of these occurrences, instead of the sole CNT contribution, would partake in establishing the WF measured for the ITO–CNT electrode (as depicted in Figure 4). Nevertheless, this increase in WF is strongly beneficial because it brings the electrode WF closer to that of the photoactive blend. Thus we anticipate a reduction in the hole–injection barrier at the anode interface, as a result of the highest occupied states of ITO–CNT lying lower than those of ITO.

A similar kind of band alignment is almost achieved in the standard cell architecture by the insertion of a layer of poly(3,4-ethylene dioxythiophene):(polystyrene sulfonic acid) (PEDOT:PSS). This polymer is used to improve the contact (and reduce the mismatch in energy level) between the ITO and the P3HT, although it is also known to shorten the device lifetime [33]. Being slightly acidic, the PEDOT:PSS is in fact able to etch the ITO and causes interface instability through indium diffusion into the polymer active layer. In our case instead, we believe that using a mat of MWCNTs as a functional buffer layer for ITO should guarantee an increase in both the charge collection and in the lifetime of the device.

**Figure 5:**
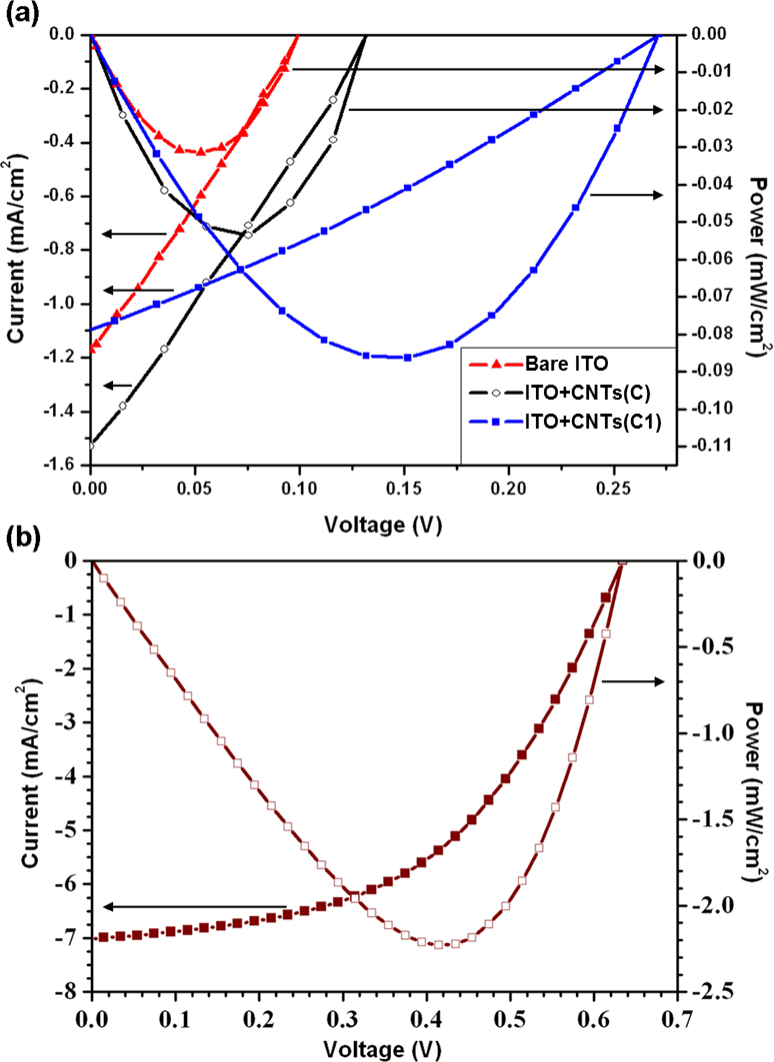
Current–voltage characteristic and output power of P3HT:PCBM solar cells: (a) Cell C and cell C1, compared to a reference cell made with bare ITO-coated glass; (b) classic ITO/PEDOT:PSS/P3HT:PCBM/Al cell manufactured in our labs.

In order to test the last statement, test organic solar cells were built with two of our CNT-enhanced anodes: sample C (whose characterization have been presented and discussed above) for cell C, and sample C1 (treated with the same CVD conditions of sample C but for a shorter time of 15 min instead of 30 min) for cell C1. The *I*–*V* curve and the output power generated by the cells made with our electrodes are reported in Figure 5a, in comparison with the data obtained for a reference cell made with a standard ITO-coated glass anode (i.e., without the addition of PEDOT:PSS). The *I*–*V* characteristic of a standard ITO/PEDOT:PSS/P3HT:PCBM/Al cell is also reported in Figure 5b, for a full understanding of the experimental results. All the numeric values are reported in Table 2, along with the respective PCEs.

**Table 2:** Characteristics of the organic solar cells: open-circuit voltage (*V*_oc_), short-circuit current density (*J*_sc_), fill factor (FF) and power conversion efficiency (η).

cell	*V*_oc_ [mV]	*J*_sc_ [mA/cm^2^]	FF [%]	η [%]

ITO/P3HT:PCBM/Al	100	−1.2	23	0.03
ITO–MWCNTs/P3HT:PCBM/Al (cell C)	140	−1.7	24	0.06
ITO–MWCNTs/P3HT:PCBM/Al (cell C1)	272	−1.1	29	0.09

As a preliminary remark, it has to be pointed out that the overall PCE of the experimental cells suffers from the lack of those beneficial effects that are acknowledged by the inclusion of a PEDOT:PSS layer between the ITO and active blend, particularly an advantageous interface morphology [34] that enables higher *J*_sc_ and fill factor (FF). On the other hand, the comparison between experimental devices made with pristine and CNT-enhanced ITO–glass demonstrates the substantial improvement that the addition of CNTs affords to the electrical properties of the electrode.

**Figure 6:**
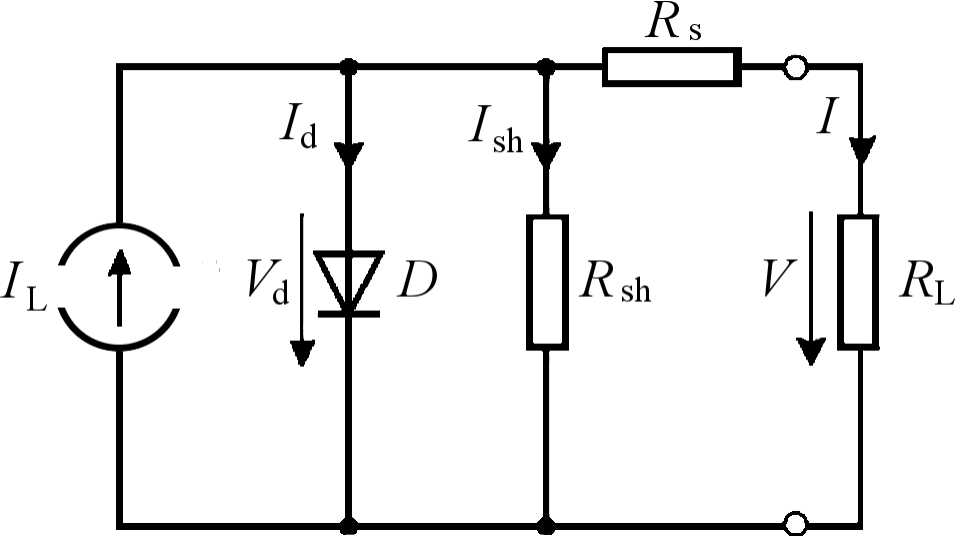
Equivalent circuit of the ideal organic solar cell.

By analyzing the *I*–*V* graphs, one readily notices how the two CNT-enhanced electrodes dramatically contribute to an increase of the open-circuit voltage (*V*_oc_) of the cell. Remarkably, in the case of cell C1, *V*_oc_ reached 272 mV, which is almost three times higher than the value measured for the reference cell made with bare ITO (~100 mV). Such a consistent improvement in *V*_oc_ is due to the optimal alignment of the energy levels between the CNT-modified ITO WF (~4.95 eV) and the P3HT HOMO (~5 eV), on the account of a fostered hole collection at the anode/polymer interface. Besides, by taking into consideration an equivalent-circuit diagram for a bulk heterojunction solar cell (Figure 6), we highlight that the CNTs could be also responsible of a quenched recombination both at the dissociation sites (e.g., donor/acceptor interfaces) and near the anode (as a result of an increase of the shunt resistor *R*_sh_), with a further positive effect on the *V*_oc_.

Moreover, we propose that our electrode could contribute to the reduction of the series resistance *R*_s_ of the cell by means of the addition of shorter and direct paths for charge collection, which are on average provided by the MWCNTs (having intrinsically a very high aspect ratio). This helps in overcoming the low mobility of the holes, now able to travel more quickly than in the pure P3HT phase, and implies a corresponding increase in *J*_sc_. In particular, the *J*_sc_ is expected to benefit from the numerous percolation paths created by the CNTs, which can effectively drive away the free carriers generated from the dissociation of the excitons at the dispersed heterojunctions.

We observe, however, that the *J*_sc_ exhibits a noteworthy 40% increase in the case of cell C, but it does not vary much for sample C1. This different behavior for two electrodes prepared with the same procedure must be explained in terms of the only parameter varied, i.e., the CVD time. Consistently with the widely known CNT growth mechanism, the shorter CVD time used for sample C1 (15 min) leads to a shorter length of the grown CNTs: as a result, we speculate that the occurrence of short circuits between the two electrodes should be less likely in this case. Hence, the *V*_oc_ is expected to augment correspondingly, while the charge collection and hence the *J*_sc_ are less enhanced by the shorter transport paths.

Conversely, the formation of an extra blocking contact (e.g., for holes at the ITO electrode) can be the reason for the small FF values found, which increases only by ~5% in the case of sample C1. This could be considered in the equivalent circuit with the insertion of a counter diode D2 or by another shunt *R*_sh_ that directly connects the two electrodes.

As already stated, the absolute efficiency of our cell is not as relevant in the present work as the comparison with the bare ITO cell is. Even without the good ohmic contact provided by PEDOT:PSS, our devices show major improvement in electric performance. In fact, the overall increase in PCE is still more noticeable when considering the lower transparency (due to the CNTs layer) and the higher resistivity (due to thermal and chemical degradation) of the treated ITO film. Nonetheless, CNT-enhanced electrodes may be used in conjunction with a layer of PEDOT:PSS to further advance the PCE of OSCs; or, once the process would be refined, they could become a suitable replacement for PEDOT:PSS, with the aim of improving the interface morphology without compromising the long-term stability of the cell. To this end, more research should be devoted to obtain a more uniform and ohmic contact between the CNTs and the P3HT. Our method could be further improved by exploring very low CVD temperatures (down to 350 °C), which have been reported as being unexpectedly suitable for CNT synthesis from Fe films [35].

## Conclusion

We presented experimental evidence of the superior electrical behavior of CNT-enhanced ITO–glass electrodes in comparison to pristine ITO ones. When implemented in experimental P3HT:PCBM solar cells, such electrodes provide a 40% increase in PCE, in spite of the slight reduction of the cell transparency. We have grown a low density carpet of MWCNTs by using a very thin film of Fe catalyst on ITO-coated glass. By investigating the effect of the growth temperature on the nanotube yield and on the ITO layer, we have selected the optimal CVD conditions for the use of such substrates as anodes for P3HT:PCBM solar cells. These process conditions address three of the biggest hindrances that affected the PCE of polymer cells made from similarly treated electrodes, because in our case (i) the sheet resistance of the electrode undergoes a limited increase during the low temperature CVD; (ii) the light transmittance of the ITO–glass does not reduce much, thanks to the low nanotube density obtained with an ultrathin (2 nm) layer of catalyst; and (iii) the occurrence of short circuits with the counter electrode is limited by the short length of the CNTs. By using this set of parameters, we built a 3D nanostructured electrode that improved the performance of the cell both in terms of *V*_oc_ (40%) and *J*_sc_ (30%).

## Experimental

MWCNTs were grown by CVD on borosilicate glass substrates coated by ITO stripes (Kintec Company, 15 Ω/sq, 100 nm thick). The substrates were cleaned by ultrasonic baths in acetone, ethanol and deionized water. Thin layers of Fe (~3 nm) were deposited as a catalyst by thermal evaporation. After the metal deposition, the substrates were loaded into a ceramic furnace for ambient-pressure CVD. The synthesis occurred in a temperature range of 500–600 °C, while a constant flow of 10% C_2_H_2_ in H_2_ (15:150 sccm) was maintained. After CVD, the substrates were analyzed by SEM and EDX (FEI - Quanta 3D 200). Transmittance values of the as-prepared electrodes were acquired with a UV–vis spectrophotometer (Shimadzu UV-2550). Nanotube morphology was also investigated by TEM (Jeol 1011 TEM).

**Figure 7:**
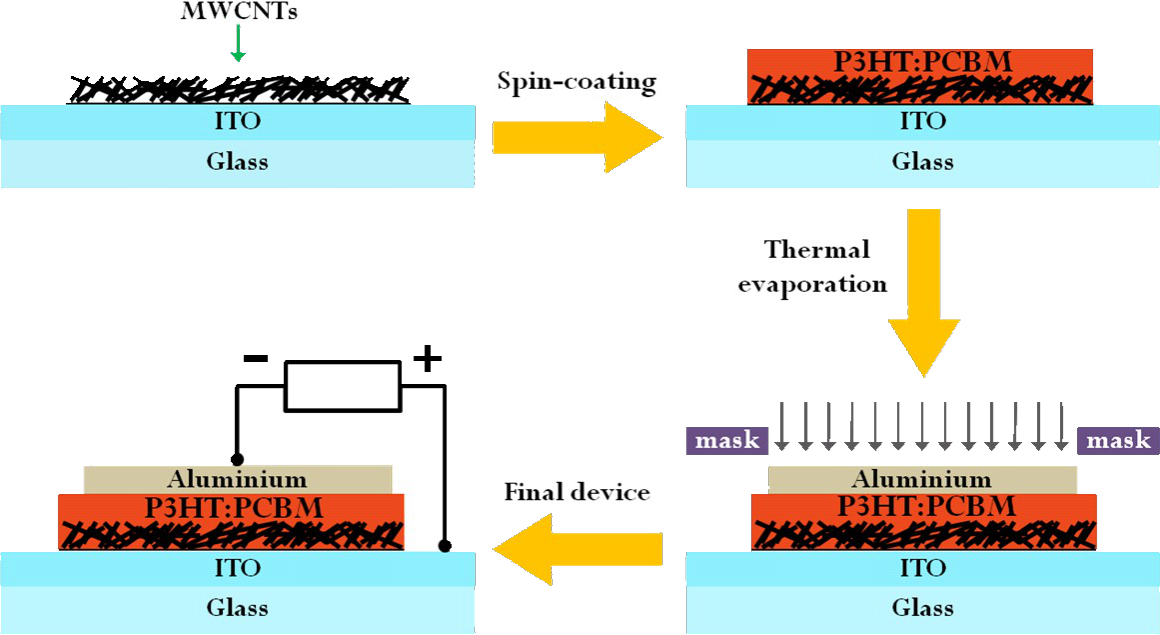
Schematics of the preparation of a P3HT:PCBM solar cell with CNT-enhanced ITO.

Bulk-heterojunction solar cells were built in a nitrogen atmosphere glovebox by using two of our CNT-enhanced ITO substrates as anodes (Figure 7). A solution (1:0.7) of regioregular poly(3-hexylthiophene) (P3HT, from Sigma-Aldrich) and phenyl-C_61_-butyric acid methyl ester (PCBM, from Solenne BV) was diluted in ortho-dichlorobenzene and spin coated at 400 rpm onto CNT-enhanced ITO-coated glass, which had been previously cleaned with acetone and isopropyl alcohol in ultrasonic baths. A 100 nm thick Al cathode was then thermally evaporated in high vacuum (~2 × 10^−6^mbar), by using a shadow mask with 3 mm wide stripes. The final device had an active area of 25 mm^2^. Reference cells with bare ITO-coated glass were also made for comparison with the same procedure. The current–voltage (*I*–*V*) characteristics under 1 sun (AM1.5G) were measured with an Agilent E5262A source meter.
